# MEANING IN LIFE AMONG THE OLD-OLD

**DOI:** 10.1093/geroni/igz038.2643

**Published:** 2019-11-08

**Authors:** Polina Ermoshkina, Eva Kahana, Boaz Kahana

**Affiliations:** 1 Case Western Reserve University, Cleveland, Ohio, United States; 2 Cleveland State University, Cleveland, Ohio, United States

## Abstract

Older adults, who view their lives as meaningful demonstrate better physical and mental health (Krause, 2007). However, the voices and the experiences of the old-old about what gives meaning to their lives have rarely been explored. This descriptive study examined key components of having meaning in life among independently living old-old migrants to the sunbelt. The sample consisted of 27 women and 18 men (N=45), with the mean age of 88.5 (SD=3.75) and the median annual income of $22,400, who participated in the Florida Retirement Study (Kahana et al., 2002). In response to the question “What gives the greatest meaning to your life at present?” family was reported as the primary source of meaning for the vast majority (40) of the participants. This is consistent with Tornstam’s (1997) theory of gerotranscendence reflected in a decreased sense of self-centeredness and greater connection to other generations. Men were more likely to list spouse as the primary source of meaning in life, followed by family, while women referred more generally to family. Health was equally important for men and women, followed by close friendships reported by 22 participants. This finding is consistent with Carstensen’s (2003) socioemotional selectivity theory suggesting that with age, the meaning of relationships changes and superficial relationships fade away. For those, who reported being very religious (7) Christian faith and attending church comprised key determinants. A transcendent, rather than materialistic view of life was illuminated by the fact that only one participant reported money as the greatest meaning in life.

